# Relationship between adjustability of grasping force and upper limb/hand function in individuals with cerebrovascular disorders

**DOI:** 10.1038/s41598-026-38384-z

**Published:** 2026-02-04

**Authors:** Tatsuya Kaneno, Katsutoshi Kawahara, Tatsuya Yabe, Takashi Tasaka, Tomoyuki Sakurai, Akihiro Sato, Kazunori Akizuki, Yoshifumi Morita

**Affiliations:** 1 Department of Occupational Therapy, Graduate School of Human Health Sciences, Tokyo Metropolitan University, Tokyo, Japan; 2 Department of Rehabilitation, Saitama Central Hospital, Saitama, Japan; 3 Department of Rehabilitation, IMS Fujimi General Hospital, Saitama, Japan; 4 Department of Occupational Therapy, Mejiro University, Saitama, Japan; 5 Department of Physical Therapy, Mejiro University, Saitama, Japan; 6 Graduate School of Engineering, Nagoya Institute of Technology, Aichi, Japan

**Keywords:** Adjustability of grasping force, Cerebrovascular disease, Rehabilitation, Upper limb/hand function, Cerebrovascular disorders, Disability

## Abstract

Activities of daily living, including grasping, holding, and pinching, are essential for independence but often become difficult for individuals with cerebrovascular disease owing to upper limb paralysis. We investigated the relationship between adjustability of grasping force (AGF), defined as the ability to adjust grasping force, and upper limb/hand functional performance. Twelve individuals with mild cerebrovascular disorders participated in this preliminary cross-sectional study. Assessments included the AGF task using iWakka, the Fugl-Meyer Assessment, Simple Test for Evaluating Hand Function, Action Research Arm Test (ARAT), and Motor Activity Log (MAL). AGF was quantified using the AGF score, calculated as the absolute error between target and actual grasping force; lower AGF scores indicate better AGF. Partial Spearman’s rank correlations, adjusted for age, revealed that AGF score (isometric section) on the less-affected side was negatively and positively correlated with ARAT and MAL (Quality of Movement) on the less-affected and more-affected sides, respectively. These findings suggest that better AGF on the less-affected side may enhance motor performance on that side but could be associated with lower perceived movement quality on the more-affected side. This highlights the complex role of the less-affected side in post-stroke function and suggests that rehabilitation strategies should consider AGF on both sides of the body.

**Supplementary Information:**

The online version contains supplementary material available at 10.1038/s41598-026-38384-z.

## Introduction

Activities of daily living, such as grasping, holding, and pinching, are essential for the performance of daily living^1^. However, they often become difficult in individuals with cerebrovascular disease owing to motor paralysis of the upper limbs and fingers caused by damage to the cerebral hemisphere. Therefore, individuals with cerebrovascular diseases are primary targets for rehabilitation. In rehabilitation, the performance of grasping, holding, and pinching in individuals with cerebrovascular disease is assessed using upper limb/hand function evaluation methods, and intervention programs are planned based on the results of these evaluation methods. In clinical practice, upper limb/hand function is commonly evaluated using the Fugl-Meyer Assessment (FMA)^2,3^, Simple Test for Evaluating Hand Function (STEF)^4^, Action Research Arm Test (ARAT)^3^, Motor Activity Log-14 (MAL)^5,6^, and Purdue Pegboard Test^7^. However, these upper limb/hand function evaluation methods only assess the maximum output required to accomplish a task, as they are based on the time required to perform a task and the degree of task accomplishment.

The ability to constantly adjust the force dynamically in response to object characteristics, such as shape, weight, and material, with less than the maximum output without dropping the grasped objects (referred to as the adjustability of grasping force [AGF]) is crucial for performing activities of daily living^8^. Previous studies have confirmed the reliability of AGF assessment using iWakka, a device developed to measure AGF^8,9^. In a study involving 41 chronic stroke survivors, in which the use of the more-affected hand was investigated, mild-to-moderate stroke survivors used the entire more-affected hand to stabilise objects (71.2%) rather than pinching with their fingers (28.8%)^10^. The AGF assessment using iWakka can evaluate a new aspect of upper limb/hand function. This method of assessment differs from the conventional maximum output evaluation because it examines fine force control during a task. The task in the AGF assessment using iWakka involves grasping the cylindrical device using the entire hand and coordinating the grasp. This is similar to the daily hand situation employed by individuals with stroke, making it easy to reflect the obtained data in daily life.

For upper limb/hand function, AGF and the ability to produce maximum output are two distinct abilities that may be associated with each other; nonetheless, the relationship between AGF and various upper limb/hand function assessments remains unclear. Evaluations and interventions are often actively conducted on the more-affected side to rehabilitate individuals with cerebrovascular disease; however, the more-affected and less-affected sides of the upper limb/hand function are impaired, highlighting the importance of approaching the less-affected side^11^. Healthy people use their upper limbs at the same level; however, individuals with cerebrovascular disease use their less-affected hands more frequently than their more-affected ones^12^. Therefore, to support the lives of individuals with cerebrovascular disease, knowledge regarding the AGF on the more-affected and less-affected sides, which is used more frequently and is closely associated with their lives, must be accumulated. If knowledge about the relationship between AGF and upper limb/hand function on the more-affected and less-affected sides could be obtained, we can evaluate and intervene with AGF based on this relationship. Therefore, in this study, we aimed to clarify the relationship between AGF and upper limb/hand functional assessments in individuals with mild cerebrovascular disease.

## Methods

### Study design

A preliminary cross-sectional design was employed in this study, in which we examined associations between AGF and upper limb/hand functional assessments in individuals with mild cerebrovascular disease during hospitalization in a convalescent rehabilitation ward.

### Participants

Participants were recruited between August 2020 and December 2023 from a rehabilitation hospital in Saitama Prefecture, Japan. All participants were in the subacute phase of stroke recovery (1–6 months post-onset) and admitted to a convalescent rehabilitation ward. The inclusion and exclusion criteria were based on a study conducted by Taub et al.^13^. Because the AGF score is measured by opening and closing the cylindrical iWakka device, participants must be able to perform active flexion and extension of the fingers to complete the task. Therefore, only individuals with relatively mild motor impairments, as confirmed using upper limb/hand function assessments, were included in this study. The inclusion criteria were as follows: (i) diagnosis of cerebrovascular disease; (ii) absence of pain or other disease in the less-affected hand that could interfere with task performance; (iii) Brunnstrom recovery stage ≥ IV in the upper limb/finger^14^; (iv) ability to voluntarily extend the wrist joint by ≥ 20° on the more-affected side; (v) ability to voluntarily extend the interphalangeal (IP) and metacarpophalangeal (MP) joints of fingers I to III fingers by ≥ 10° on the more-affected side; (vi) ability to maintain a stable sitting position; and (vii) ability to understand the examiner’s verbal instructions. The exclusion criteria encompassed: (i) having performed similar tasks in the past; (ii) current or past history of orthopedic or neurological disease on the less-affected side that could interfere with daily life; (iii) significant cognitive impairment; (iv) considerable impairment, including aphasia, attention disorder, and unilateral spatial neglect; (v) inability to see the monitor because of significant visual impairment (hemianopia, diplopia, decreased visual acuity); and (vi) severe complications that were not medically controlled.

The informed consent was obtained, by explaining the purpose and content of this study orally and in writing to individuals with cerebrovascular disease who met the inclusion criteria. Twelve people who agreed to participate in this study were selected as the participants. The study was performed in accordance with the Declaration of Helsinki and was approved by the Ethics Review Committees of Mejiro University (approval no. 19–045) and Tokyo Metropolitan University (approval no. 22046).

### Measurement items

#### Basic data

Basic data such as age, sex, dominant hand, more-affected side, period from the onset, grip strength, Mini-Mental State (MMS) score^15^, and Functional Independence Measure (FIM)^16^ were collected. The participants’ dominant hand was determined using the Edinburgh Handedness Inventory, with a laterality index of ≥ 0 indicating right-handedness and a laterality index of < 0 indicating left-handedness^17^.

#### iWakka

**Fig. 1:**
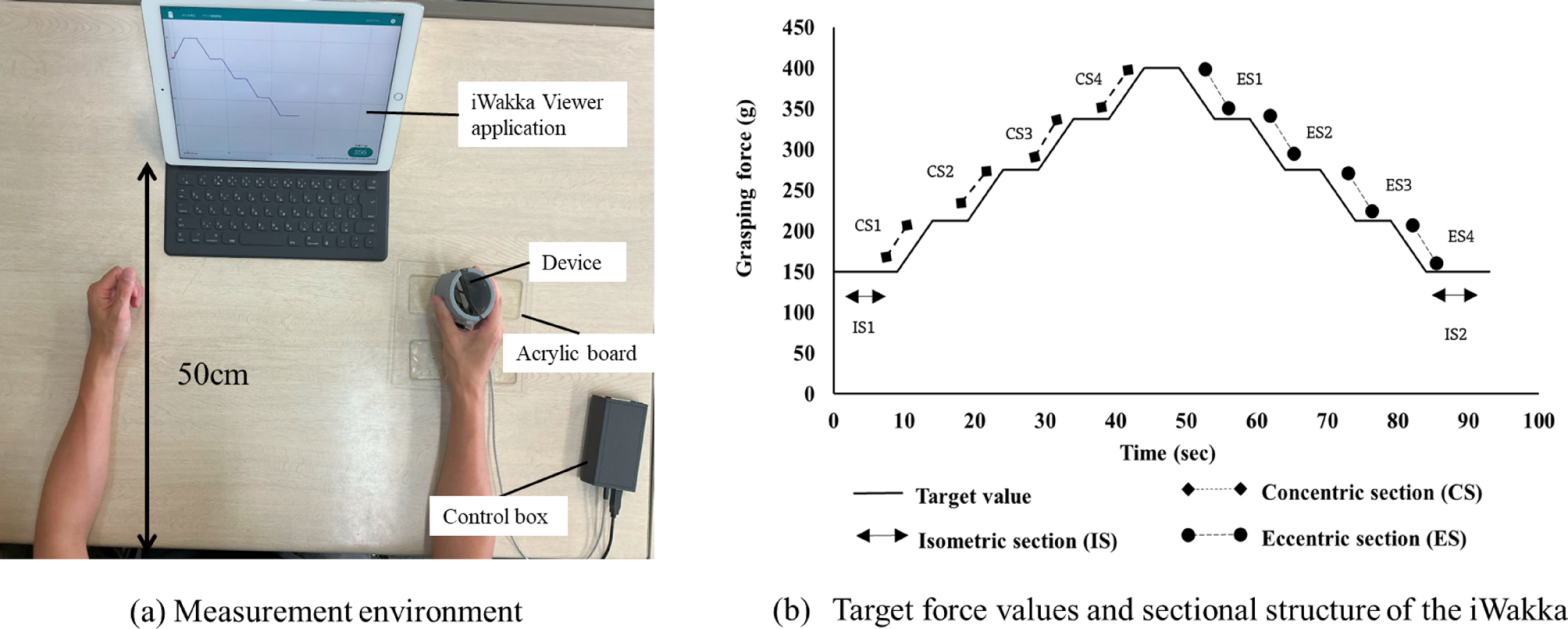
AGF assessment using iWakka. (**a**) The iWakka device consists of a longitudinally split PVC pipe with a hinge and crossed leaf springs to measure grasping force changes. Strain is recorded and displayed in real time on a tablet. The device was placed on an acrylic board with both elbows on the table and the forearms in the neutral position to eliminate the influence of the upper limbs and keep the height of both shoulders constant. (**b**) The AGF score was calculated for each section, namely, the isometric (IS1–IS2), concentric (CS1–CS4), and eccentric (ES1–ES4) sections, as the average absolute error between the target and actual grasping force over a 5-second analysis window. For the isometric section in particular, only the initial and final intervals (IS1 and IS2) were used in the analysis to ensure that the data were collected without influence from the preceding or subsequent concentric and eccentric phases. AGF, Adjustability of grasping force; PVC, polyvinyl chloride.

This study evaluated AGF using the iWakka (Nagoya Institute of Technology), which comprised a grasping device, a control box, an iPad (Apple Inc., Cupertino, CA, USA), and the iWakka Viewer application installed on the iPad. The cylindrical device consisted of two parts made by cutting a polyvinyl chloride pipe (height: 80 mm, diameter: 65 mm) lengthwise into approximately equal halves. These two halves were joined by a hinge along one side of the cut, forming a clamshell-like structure that could be opened and closed. Inside the device, leaf springs were attached in a crossed configuration, and the strain generated by opening and closing the device was measured using a strain gauge. The strain gauge signal was amplified and converted to a digital signal via an amplifier circuit and an analogue-to-digital converter (ADC) built into the control box. This digital voltage data was then transmitted to an iPad via Bluetooth. The iPad used the iWakka Viewer application to convert the voltage data into grasping force values, which were displayed in real time as a graph. The sampling frequency of the measurement was 10 Hz. No additional hardware or software filtering was applied (Fig. 1a). The participants adjusted their grasping power based on the target value displayed on the monitor of the iPad. The maximum target value was 400 g, which changed step-by-step at regular intervals and was displayed as it flowed toward the left edge of the monitor over time. The AGF score was quantitatively evaluated by calculating the absolute error between the target value and the actual grasping force. The use of absolute values allowed us to focus on the overall accuracy of force control by quantifying the magnitude of deviation, regardless of direction. Importantly, AGF refers to the conceptual ability to adjust grasping force in accordance with task demands, whereas the AGF score is a numerical indicator of this ability. Therefore, a lower AGF score indicates better AGF, meaning more accurate grasping force adjustment.

There were three types of sections, namely, (i) the isometric section, where the grasping force was maintained at a constant level over time; (ii) the concentric section, where the grasping force was gradually strengthened; and (iii) the eccentric section, where the grasping force was gradually weakened (Fig. 1b). A previous study involving healthy older adults reported that the AGF differed depending on each section, requiring different adjustment methods for each interval^18^. To further examine the characteristics of this relationship between AGF and upper limb/hand functional assessments in individuals with mild cerebrovascular disorders, AGF scores were calculated not only for the total sections but also separately for each section. The AGF score for each section, the isometric (IS1–IS2), concentric (CS1–CS4), and eccentric (ES1–ES4) sections, was calculated as the average absolute error between the target and actual grasping force over a 5-second analysis window (Fig. 1b). For the isometric section in particular, only the initial and final intervals (IS1 and IS2) were used in the analysis to ensure that the data were collected without influence from the preceding or subsequent concentric and eccentric phases.

Based on a previous study^8^, the measurement environment was set as follows: the distance between the table and the body was approximately 10 cm, and the monitor of iPad was placed 50 cm from the edge of the table. The device was placed on an acrylic board with both elbows on the table and the forearms in the neutral position to eliminate the influence of the upper limbs and keep the height of both shoulders constant. A practice session using a target value different from that of the evaluation tasks was conducted to ensure that participants could perform the iWakka task reliably and consistently before data collection. After performing the practice tasks, we decided to perform the evaluation tasks in the same environmental settings as the practice tasks. The evaluation tasks were performed in two trials, and the average value was used as the measured value. Evaluation was conducted on the less-affected side and subsequently on the more-affected side.

#### Fugl-Meyer Assessment (FMA)

The FMA is an evaluation method developed to determine the degree of paralysis in individuals with stroke, and its reliability and validity have been confirmed^2^. The FMA includes 33 items, with 18 items for the upper limbs, five for the wrist joints, seven for the fingers, and three for coordination. The score is given out of 66 points based on the voluntary movements of the upper limbs and fingers, with a higher score indicating better motor ability.

#### Simple Test for Evaluating Hand Function (STEF)

The STEF is an evaluation method for assessing the upper extremity function, and its reliability and validity have also been validated^4^. The STEF comprises 10 subtests, and each subtest is scored based on the time it takes to transport items of varying weights and materials. The total score is given out of 100 points, with a higher total score suggesting a higher function.

#### Action Research Arm Test (ARAT)

The ARAT is an evaluation method for upper limb/finger function that has been internationally confirmed as reliable and valid^3^. The ARAT includes 19 items: six for grasping, four for gripping, six for pinching, and three for gross movement. Each item is scored on a 4-point ordinal scale ranging from 0 to 3, with a total possible score of 57. Higher scores indicate better upper extremity function.

#### Motor Activity Log-14 (MAL)

The MAL is used to evaluate the frequency and quality of use of more-affected hands in daily life^5,6^. The amount of use (AOU) and quality of movement (QOM) of the upper limbs are subjectively evaluated using a six-item method for 14 tasks performed in daily life, and the average value for each item is calculated. A higher MAL score indicates higher frequency and quality of use of the more-affected hand.

### Data analysis

A Spearman’s rank correlation coefficient analysis was performed to investigate the relationship between AGF score and each item (age, FMA, STEF, ARAT, MAL). Given prior evidence that the AGF score is influenced and tends to increase with age, reflecting poorer AGF^19^, we conducted a partial Spearman’s rank correlation analysis adjusted for age. Statistical analyses were performed using IBM SPSS Statistics version 27, and statistical significance was set at 5%.

## Results

### Basic characteristics of the participants

In total, six men and six women with a median age of 67.5 years (interquartile range [IQR]: 59.0–74.8) were included in this study (Table 1, Supplementary Table S1). Of the 12 participants, 11 were right-handed and one was left-handed. Regarding the more-affected side, four and eight had left-sided and right-sided impairments, respectively. The median period from onset was 78.0 days (IQR: 59.0–93.0). Median grip strength was 8.7 kg (IQR: 5.8–18.5) on the more-affected side and 21.8 kg (IQR: 16.2–27.8) on the less-affected side. The median MMS and FIM scores were 28.5 (IQR: 26.8–30.0) and 118.5 (IQR: 108.5–124.0) points, respectively. Table 1 shows the results of FMA, STEF, ARAT, and MAL assessments. The median AGF score on the more-affected side was 16.4 g in the total sections (IQR: 8.8–26.9), 9.1 g in the isometric section (IQR: 6.0–15.7), 14.7 g in the concentric section (IQR: 7.7–30.0), and 18.0 g in the eccentric section (IQR: 10.6–30.8). The median AGF score on the less-affected side was 9.3 g in the total sections (IQR: 5.2–21.0), 5.6 g in the isometric section (IQR: 3.0–13.4), 9.5 g in the concentric section (IQR: 5.9–20.9), and 11.2 g in the eccentric section (IQR: 5.7–16.4).

**Table 1:** Basic characteristics of the participants.

	*n*	Median (IQR)	Min/Max
Age (years)		67.5 (59.0–74.8)	52/87
Sex, male/female	6/6		
Dominant hand, left/right	1/11		
More-affected side, left/right	4/8		
Period from onset (days)		78.0 (59.0–93.0)	42/132
Grip strength			
More-affected side		8.7 (5.8–18.5)	3/36.2
Less-affected side		21.8 (16.2–27.8)	5/39.6
MMS		28.5 (26.8–30.0)	22/30
FIM		118.5 (108.5–124.0)	64/126
FMA		60.5 (52.5–66.0)	36/66
STEF			
More-affected side		80.0 (39.8–86.3)	2/99
Less-affected side		91.0 (86.8–94.0)	66/99
ARAT			
More-affected side		51.5 (37.5–57.0)	2/57
Less-affected side		57.0 (57.0–57.0)	26/57
MAL (AOU)		4.3 (2.2–5.0)	0/5
MAL (QOM)		3.6 (2.7–4.3)	0/5
AGF score on the more-affected side (g)			
Total sections		16.4 (8.8–26.9)	5.2/126.8
Isometric sections		9.1 (6.0–15.7)	3.0/47.1
Concentric sections		14.7 (7.7–30.0)	5.5/123.8
Eccentric sections		18.0 (10.6–30.8)	5.8/169.7
AGF score on the less-affected side (g)			
Total sections		9.3 (5.2–21.0)	3.8/74.3
Isometric sections		5.6 (3.0–13.4)	2.5/58.0
Concentric sections		9.5 (5.9–20.9)	4.3/82.6
Eccentric sections		11.2 (5.7–16.4)	4.0/82.6

IQR: Interquartile range, MMS: Mini-Mental State, FIM: Functional Independence Measure, FMA: Fugl-Meyer Assessment, STEF: Simple Test for Evaluating Hand Function, ARAT: Action Research Arm Test, MAL: Motor Activity Log-14, AOU: amount of use, QOM: quality of movement.

### Relationship between the AGF and upper limb/hand function evaluation

Table 2 shows the relationship between the AGF score and upper limb/hand function evaluation. On the more-affected side, AGF scores (total, isometric, and concentric sections) showed a moderate-to-strong positive correlation with age, indicating that AGF declines with increasing age. Similarly, the AGF scores on the less-affected side (total, isometric, concentric, and eccentric sections) also displayed moderate-to-strong positive correlations with age. In contrast, AGF scores on the less-affected side (total, isometric, and eccentric sections) showed a moderate-to-strong negative correlation with STEF on the same side, indicating that better AGF (lower AGF score) was associated with better functional performance. Likewise, the isometric section AGF score on the less-affected side showed a moderate negative correlation with the ARAT on the same side, also indicating that better AGF (i.e., lower AGF score) was associated with better functional performance. Furthermore, a significant positive correlation was observed between the AGF score (isometric section) on the less-affected side and the MAL (QOM) score (*r* = 0.61, *p* = 0.04), suggesting that individuals with poorer AGF (higher AGF scores) on the less-affected side may perceive better quality of movement in the more-affected limb.

**Table 2:** Relationship between AGF score and upper limb/hand function evaluation.

	Age	FMA	STEF (more-affected)	STEF (less-affected)	ARAT (more-affected)	ARAT (less-affected)	MAL (AOU)	MAL (QOM)
AGF score on the more-affected side (g)								
Total sections	0.71*	− 0.13	− 0.26	− 0.30	− 0.28	− 0.24	− 0.21	− 0.06
Isometric sections	0.62*	0.14	− 0.03	− 0.17	− 0.04	− 0.30	0.01	0.15
Concentric sections	0.71*	− 0.13	− 0.26	− 0.30	− 0.28	− 0.24	− 0.21	− 0.06
Eccentric sections	0.56	− 0.12	− 0.02	− 0.18	− 0.19	− 0.51	− 0.11	0.07
AGF score on the less-affected side (g)								
Total sections	0.71**	0.08	0.01	− 0.68*	− 0.04	− 0.57	0.05	0.26
Isometric sections	0.69*	0.38	0.33	− 0.70*	0.36	− 0.64*	0.46	0.61*
Concentric sections	0.64*	0.14	0.05	− 0.56	0.04	− 0.50	0.08	0.34
Eccentric sections	0.73**	0.15	0.11	− 0.59*	0.04	− 0.57	0.11	0.30

Spearman’s rank correlation coefficients (r) are shown between AGF scores for each section (total, isometric, concentric, and eccentric) and age, as well as upper limb functional measures (FMA, STEF, ARAT, and MAL). * = *p* < 0.05, ** = *p* < 0.01. Lower AGF scores indicate better AGF.

FMA: Fugl-Meyer Assessment, STEF: Simple Test for Evaluating Hand Function, ARAT: Action Research Arm Test, MAL: Motor Activity Log-14, AOU: amount of use, QOM: quality of movement, AGF: adjustability of grasping force.

### Relationship between AGF and upper limb/hand function evaluation after adjusting for age

Table 3 shows the age-adjusted relationship between AGF scores and upper limb/hand function evaluations. The AGF score (isometric section) on the less-affected side was negatively correlated with the ARAT score on the same side (*r* = − 0.64, *p* = 0.04), indicating that better AGF (lower AGF score) was associated with better upper limb function. Additionally, a significant positive correlation was found between the AGF score (isometric section) on the less-affected side and the MAL (QOM) score (*r* = 0.65, *p* = 0.03), suggesting that individuals with poorer AGF (higher AGF scores) may perceive better movement quality in the more-affected limb.

**Table 3:** Relationship adjusted for age between AGF score and upper limb/hand function evaluation.

	FMA	STEF (more-affected)	STEF (less-affected)	ARAT (more-affected)	ARAT (less-affected)	MAL (AOU)	MAL (QOM)
AGF score on the more-affected side (g)							
Total sections	− 0.13	− 0.34	0.29	− 0.47	− 0.06	− 0.45	− 0.31
Isometric sections	0.21	− 0.02	0.38	− 0.11	− 0.17	− 0.10	0.02
Concentric sections	− 0.13	− 0.34	0.29	− 0.47	− 0.06	− 0.45	− 0.31
Eccentric sections	− 0.11	− 0.01	0.29	− 0.28	− 0.44	− 0.23	− 0.06
AGF score on the less-affected side (g)							
Total sections	0.17	0.04	− 0.40	− 0.13	− 0.55	− 0.08	0.15
Isometric sections	0.58	0.47	− 0.47	0.41	− 0.64*	0.50	0.65*
Concentric sections	0.22	0.08	− 0.24	− 0.01	− 0.43	− 0.02	0.27
Eccentric sections	0.27	0.19	− 0.23	− 0.02	− 0.55	0.01	0.22

Spearman’s rank correlation coefficients adjusted for age (r) are shown between AGF scores for each section (total, isometric, concentric, and eccentric) and upper limb functional measures (FMA, STEF, ARAT, and MAL). * = *p* < 0.05, ** = *p* < 0.01. Lower AGF scores indicate better AGF.

FMA: Fugl-Meyer Assessment, STEF: Simple Test for Evaluating Hand Function, ARAT: Action Research Arm Test, MAL: Motor Activity Log-14, AOU: amount of use, QOM: quality of movement, AGF: adjustability of grasping force.

## Discussion

In this exploratory study, we aimed to clarify whether a relationship exists between AGF and upper limb/hand functional assessments in individuals with mild cerebrovascular disorders. Our findings revealed that the AGF score (isometric section) on the less-affected side was negatively associated with ARAT scores on the less-affected side. In contrast, the AGF score (isometric section) on the less-affected side was positively associated with MAL (QOM) on the more-affected side. These findings suggest that poorer AGF (higher AGF scores) on the less-affected side is associated with lower objective functional performance on the same side and with higher subjective ratings of movement quality of the more-affected upper limb. This pattern does not indicate a reversal of motor ability between limbs but rather differences in the functional roles of the hands between the less- and more-affected sides.

In a previous study^18^, the average AGF score for the dominant hand of healthy older participants was 8.9 ± 4.2 g in the total section (95% confidence interval (CI): 7.5, 10.3), 5.8 ± 3.1 g in the isometric section (95% CI: 4.8, 6.9), 8.4 ± 3.9 g in the concentric section (95% CI: 7.2, 9.7), and 12.0 ± 7.0 g in the eccentric section (95% CI: 9.7, 14.3). Kaneno et al.^8^ examined the absolute reliability of the AGF score using Bland–Altman analysis and identified the limits of agreement (LOA) as − 2.8 to 4.4 g and − 2.6 to 3.9 g for the dominant and non-dominant hands, respectively. In contrast, our study results indicate that the AGF score on the more-affected side exceeded these LOA ranges, suggesting that individuals with cerebrovascular disease had poorer AGF (higher AGF scores) than healthy older adults did. Previous studies have reported that individuals with stroke exhibit discrepancies between perceived and actual motor output^20^. Such discrepancies may lead to differences between target force levels and the force values actually measured, thereby potentially affecting AGF on the more-affected side. Additionally, because the AGF score is measured by opening and closing the cylindrical iWakka, the stage of grasp coordination cannot be reached if no flexion and extension movements of the fingers are produced, limiting the accurate AGF measurement. To address these issues, the criteria for participant inclusion and exclusion were based on those outlined by Taub et al.^13^. Data were collected from participants who demonstrated flexion and extension movements of the fingers and could adjust their grasping strength. Participants in our study exhibited a relatively mild degree of disability based on the results of upper limb/hand function evaluation. However, despite the mild motor paralysis, a decline in AGF (higher AGF scores) was observed, underscoring the importance of interventions for AGF in individuals with cerebrovascular disease.

Interestingly, AGF score across all sections on the less-affected side did not differ from that in healthy older adults^18^, suggesting that it might have an AGF equivalent to that of healthy older participants, studies have shown that functional decline on the less-affected side in individuals with cerebrovascular disease is more likely to affect the distal muscles than the proximal muscles^21,22^. Therefore, continuous data collection is necessary to verify the validity of the results obtained in this study.

The findings of this study indicated that the AGF score on the more-affected side (total, isometric, and concentric sections) and the less-affected side (total, isometric, concentric, and eccentric sections) showed a moderate-to-strong positive correlation with age. This aligns with previous research on healthy participants, which reported higher AGF scores, reflecting poorer AGF, in older adults than in younger participants, and that AGF was more affected by age^19^. These results suggest that AGF declines with age on both sides in individuals with cerebrovascular disease, as reflected by increased AGF scores, mirroring trends observed in healthy participants.

A partial Spearman’s rank correlation analysis, adjusted for age, revealed a significant positive correlation between the AGF score on the less-affected side (isometric section) and the MAL (QOM) score. Specifically, the results suggest that better AGF on the less-affected side (lower AGF scores) was associated with reduced quality of use of the more-affected hand in daily life. Notably, healthy individuals typically use both upper limbs equally in daily activities. However, individuals with cerebrovascular disease tend to rely more on the less-affected hand than the more-affected hand^12^. Even among individuals with mild cerebrovascular disease, as in the present study, those with relatively greater difficulty using the more-affected hand in daily life may become increasingly dependent on the less-affected side. Consequently, the less-affected hand is used more frequently in daily activities, providing more opportunities to engage in tasks requiring AGF, whereas the more-affected hand is used less, potentially leading to a decline in its perceived quality of use. Under these conditions, the AGF of the less-affected side may be relatively preserved against age-related decline, which may have influenced the results of this study. Contrary to earlier reports suggesting a decline in finger function on the less-affected side in individuals with severe paralysis due to cerebrovascular disease^23^, our study revealed a significant positive correlation between the AGF score on the less-affected side (isometric section) and the MAL (QOM) score on the more-affected side; that is, better AGF (lower AGF score) on the less-affected side was associated with lower MAL(QOM) scores on the more-affected side. This suggests that AGF on the less-affected side may be relatively preserved even in participants with greater impairment. These findings imply that individuals with mild cerebrovascular disease and relatively lower movement quality on the more-affected side may benefit from therapeutic strategies that leverage the relatively preserved ability to adjust grasping force, as quantified using AGF scores, on the less-affected side. Conversely, those who maintain a higher quality of use of the more-affected hand may require interventions to address age-related AGF decline on the less-affected side. AGF is not a direct indicator of functional ability, but rather a performance-based measure reflecting one aspect of motor control; however, these findings suggest that evaluating AGF on the less-affected side may offer complementary insights depending on the severity of motor impairment.

The AGF score on the less-affected side (isometric section) was negatively correlated with the ARAT on the same side, indicating that individuals with better AGF (lower AGF scores) tended to demonstrate better upper limb function, as measured using the ARAT. Traditionally, clinical assessments of upper limb/hand function evaluate task performance based on the time required for or the achievement level of task completion. Conversely, AGF evaluation using the iWakka is focused on examining the ability to adjust grasping force during task execution. Consequently, the AGF assessed with iWakka represents a skill distinct from the upper limb/hand function assessments commonly used in clinical settings. Therefore, it was expected that no correlations would be observed between these measures. However, the results of this study, utilising age-adjusted partial correlation analysis, revealed that the AGF on the less-affected side was negatively correlated with ARAT on the same side. This suggests that although AGF and upper limb function are independent factors, they may influence each other. No significant correlation was observed between AGF and certain clinical assessments, including the FMA or STEF, likely owing to the differing characteristics and measurement constructs of each test. The FMA is used to assess the presence of voluntary movement patterns and is designed to evaluate motor recovery in individuals with severe impairments; however, it does not involve object manipulation or assess the AGF. In contrast, the STEF is focused on fine object manipulation with time-based scoring and is primarily applicable to individuals with very mild impairments, which may have resulted in a ceiling effect in our relatively mildly impaired sample. The ARAT, however, incorporates both gross motor and object-based tasks, offering a broader range of assessment and potentially explaining the observed moderate negative correlation with AGF score. These differences in test content and sensitivity may account for the variability in correlations between AGF and clinical assessments observed in this study.

Age-adjusted correlation analysis further revealed a significant correlation with the AGF score, particularly in the isometric section. A previous study involving community-dwelling healthy older individuals reported that AGF scores were significantly lower in the isometric section than in other intervals, indicating better AGF in this section^18^. Unlike the concentric and eccentric sections, where participants must adjust their grasping force in real time in response to continuously changing target values, the isometric section requires them to maintain a constant force against an unchanging target. As such, it is considered a relatively easier adjustment section. Therefore, individuals who exhibit large errors even in this relatively simpler section may have poorer AGF, as reflected by higher AGF scores. This likely explains why the isometric section is more sensitive in detecting severe AGF decline and why correlations with functional measures were most prominent in this section.

This study has some limitations that warrant consideration. First, the sample size was limited to 12 participants, which restricts the generalizability of the findings. Additionally, only individuals with relatively mild paralysis capable of voluntary finger movements using the iWakka device were included, restricting the applicability of the results to mildly impaired individuals with cerebrovascular disease. Future studies should include participants with a broader range of motor impairments to comprehensively examine AGF across different severity levels. Second, the AGF score used in this study was derived from the absolute error between the target and actual grasping force. While this approach allowed us to quantify overall control accuracy, it does not capture the direction of deviation (that is, over- or under-compensation). Incorporating signed error values in future analyses may provide deeper insights into the nature of grasping force control errors. Third, given that this was a cross-sectional study using correlational analysis, causal relationships between AGF and upper limb/hand function could not be established. To understand the clinical relevance and potential impact of AGF, longitudinal and interventional studies are needed. Specifically, it remains unclear whether enhancing AGF leads to improved motor performance or daily function. Fourth, while the AGF setup is simple and has potential for use in clinical settings, the optimal timing and frequency of AGF assessment remain unclear. We did not determine specific intervals for AGF testing; however, incorporating it into regular monthly rehabilitation evaluations may help monitor changes in motor control and detect early signs of functional decline. Further studies should explore the most effective periodicity for AGF testing. Finally, although a positive correlation was observed between AGF on the less-affected side and the quality of movement on the more-affected side (MAL-QOM), we do not propose AGF as a direct measure of functional ability. Rather, AGF is a performance-based research measure reflecting one aspect of motor control and should not be interpreted as a standalone clinical outcome. Additional studies are needed to clarify its relationship with real-world functional outcomes.

## Conclusions

This preliminary study demonstrated that AGF is associated with upper limb functional performance in individuals with mild cerebrovascular disease. Specifically, better AGF on the less-affected side was linked to enhanced functional ability on that side, yet paradoxically, to lower perceived quality of use on the more-affected side. These findings underscore the importance of assessing both upper limbs in post-stroke rehabilitation and suggest that AGF evaluation may provide complementary insights into motor control adaptations. Further research with larger and more diverse samples is warranted to validate these findings and explore the clinical utility of AGF assessment in rehabilitation planning.

## Supplementary Information

Below is the link to the electronic supplementary material.

Supplementary Material 1

## Data Availability

The data that support this study’s findings are available from the corresponding author on reasonable request. The data are not publicly available due to ethical restrictions.
